# Draft Genome Sequence of “*Candidatus* Nardonella dryophthoridicola” Strain NARMHE1, Endosymbiont of Metamasius hemipterus (Coleoptera, Curculionidae, Dryophthorinae)

**DOI:** 10.1128/mra.00738-22

**Published:** 2022-10-31

**Authors:** Luciano Palmieri, Ronaldo Pavarini, Prashant P. Sharma

**Affiliations:** a Department of Integrative Biology, University of Wisconsin-Madison, Madison, Wisconsin, USA; b Faculdade de Ciências Agrárias do Vale do Ribeira, Campus de Registro, São Paulo State University-UNESP, Registro, São Paulo, Brazil; Indiana University, Bloomington

## Abstract

Here, we report the draft genome and annotation of “*Candidatus* Nardonella dryophthoridicola” strain NARMHE1, obtained via Oxford Nanopore sequencing of the ovaries of its host, the weevil Metamasius hemipterus, from a population from southeast Brazil.

## ANNOUNCEMENT

*Metamasius* (family, Curculionidae; subfamily, Dryophthorinae) harbors obligate endosymbiont *Nardonella*, a group of *Gammaproteobacteria* that have cospeciated with their hosts (1–3). Metamasius hemipterus is a pest on sugarcane and other crops and has invasive potential, threatening agriculture (4–7). However, genomic sampling of *Nardonella* remains limited, particularly for host species that exhibit invasive potential. Here, we generate the genome of the *Nardonella* associated with M. hemipterus, found on cultivated Bactris gasipaes (Arecaceae).

M. hemipterus adults were collected (Pariquera-açu, São Paulo, Brazil; −24.608873, −47.896800) using scent bait traps (8). Larvae were extracted from stems and fixed in ethanol. To detect *Nardonella* presence, 15 females were dissected, and their midguts and ovaries were separated. We also dissected gut tissues of 15 larvae. Samples were immersed in 2% bleach for 60 s and dissected in 1× phosphate-buffered saline (PBS). DNA extractions were performed using Qiagen DNeasy blood and tissue kit following the manufacturer’s protocol with modifications (overnight proteinase K incubation, two double-distilled water [ddH_2_O; 56°C] elutions, and 10 min final incubation). The DNA concentration was verified with Qubit double-stranded DNA (dsDNA) high-sensitivity (HS) assay kit (Life Technologies). Samples were prepared according to Celero PCR workflow with enzymatic fragmentation (Tecan Genomics). The quality and quantity of the finished libraries were assessed with Agilent TapeStation (Agilent) and Qubit dsDNA HS assay kit. Sequencing was performed using Illumina NovaSeq6000 (2×150 bp).

**FIG 1:**
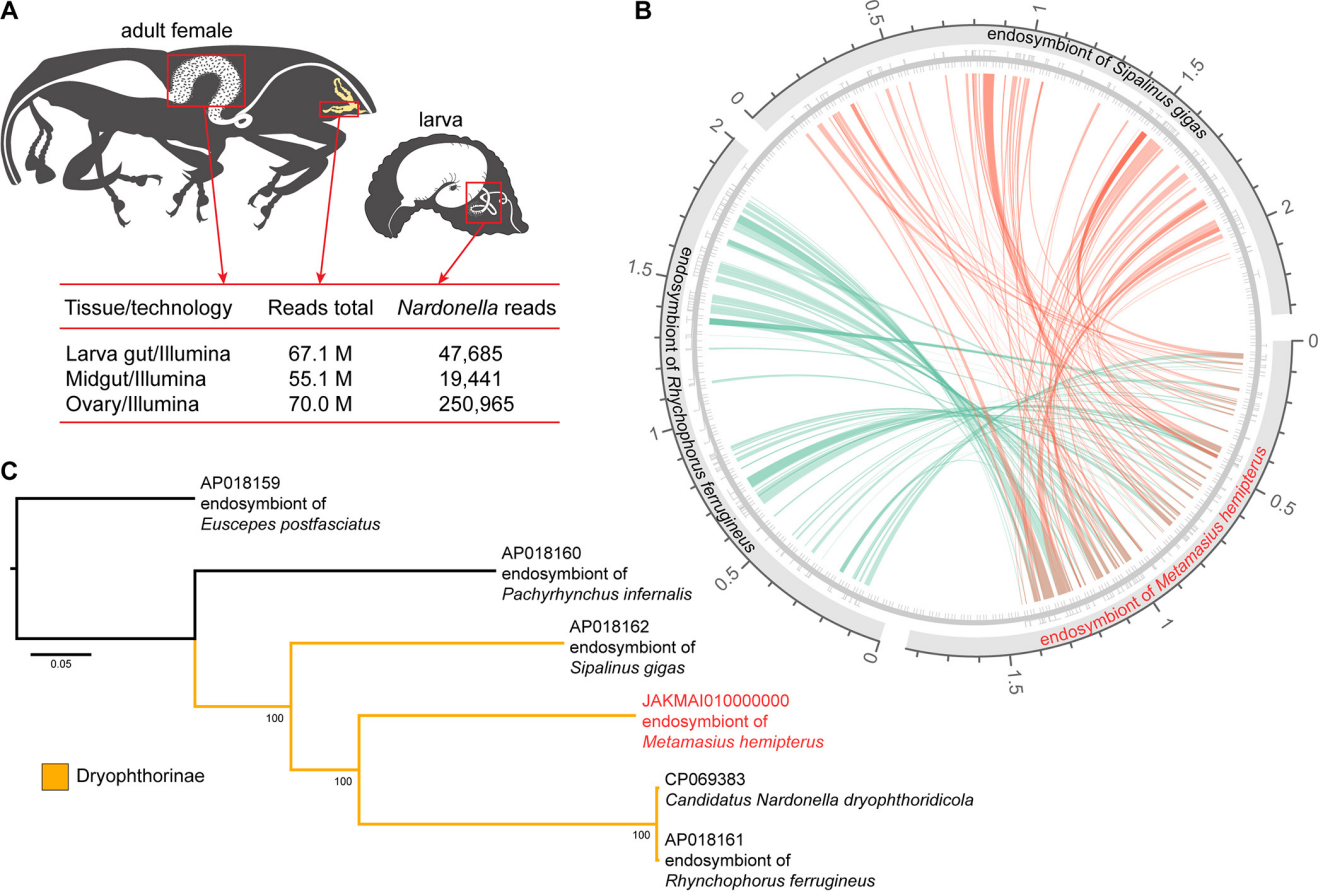
“*Candidatus* Nardonella dryophthoridicola” strain NARMHE1 draft genome assembly. (A) Determination of the most suitable tissue for recovering symbiont sequences. (B) Synteny circus plot showing rearrangement of some relevant genes on resulting draft genome compared with other Dryophthorinae endosymbiont *Nardonella* genomes. (C) Maximum-likelihood tree constructed on RAxML with all available *Nardonella* genomes. GenBank accession numbers of the sequences used are AP018159, endosymbiont of Euscepes postfasciatus; AP018160, endosymbiont of Pachyrhynchus infernalis; AP018161, endosymbiont of Rhynchophorus ferrugineus; AP018162, endosymbiont of Sipalinus gigas; CP069383, “*Candidatus* Nardonella dryophthoridicola”; and JAKMAI010000000, endosymbiont of Metamasius hemipterus (this study).

Raw reads (host and symbiont) were queried in BLASTn (9, 10) to identify sequences of *Nardonella* against the NCBI genome database (E value cutoff, 10^−6^; see supplemental information for program options). Sequences of interest were extracted using Seqtk1.3 (https://github.com/lh3/seqtk). Ovaries presented 5× more *Nardonella* sequences (Fig. 1A) and were selected for sequencing using Oxford Nanopore Technologies. No shearing/fragmentation was performed on input DNA (LSK110 kits were used for library preparation and run on two GridION-MinION flow cells; high-accuracy base calling min_qscore was 9). Long reads were processed using the same filtering procedures described for Illumina (Table 1). Long reads were deduplicated with BBMap version 38.94 (https://sourceforge.net/projects/bbmap/); sequences shorter than 130 bp were removed with Filtlong version 0.2.0.1 (https://github.com/rrwick/Filtlong). The 30,930-bp-long reads were assembled twice independently using Canu version 2.2 (11) and Flye version 2.8.3 (12). Final contigs for each assembly were corrected using unfiltered long reads with Medaka version 1.6.0 (https://github.com/nanoporetech/medaka). Additional polishing using short reads was made with Polypolish version 0.4.3 (13), with a further long-read polishing made with poLCA version 4.0.6 (14). To improve contiguity, we used quickmerge version 0.3 (15). The final assembly was composed of seven contigs (Table 1). Scaffolding was performed using homology between contigs and reference genomes. Potential misassembles were corrected with RagTag version 2.1.0 (https://github.com/malonge/RagTag). After correction, we used reference protein sequences of the Rhynchophorus ferrugineus endosymbiont, GenBank accession no. AP018161, to orient contigs. Stretches of 100 “Ns” were placed between adjacent sequences to indicate gap regions. The final genome was annotated using PGAP version 6.0 (16) (Table 1). Although some genes are incomplete, there was high similarity of genetic composition to other *Nardonella* genomes (Fig. 1B). To ascertain identification, we aligned our contigs to other *Nardonella* genomes with Mauve version 2.4.0 (17). A maximum-likelihood (ML) tree was inferred with RAxML version 8.2.11 (18) (GTR+gamma; 1,000 bootstraps). NARMHE1 formed a strongly supported clade with other Dryophthorinae endosymbionts (Fig. 1C). The positioning of *Nardonella* strains on the phylogeny emulates their dryophthorid hosts (19), suggesting a coevolutionary process (20).

**TABLE 1:** Combined assembly summary

Characteristic	Value	GenBank accession no.
Long-read characteristics		
No. of reads after deduplication	30,930	
Mean read length (bp)	883	
Longest read length (bp)	14,328	
Shortest read length (bp)	135	
*N*_50_ (bp)	3,170	
Contig characteristics (no. of bp)		
Contig 1	12,016	JAKMAI010000001
Contig 2	28,356	JAKMAI010000002
Contig 3	16,370	JAKMAI010000003
Contig 4	6,418	JAKMAI010000004
Contig 5	12,133	JAKMAI010000005
Contig 6	13,069	JAKMAI010000006
Contig 7	91,945	JAKMAI010000007
Genome characteristics		
Size (bp)	180,307	
GC content (%)	19.80	
No. of genes	199	
No. of CDSs^*a*^	144	
No. of RNAs	36	
No. of pseudogenes	19	
Complete rRNAs	5S, 16S, 23S	

a CDS, coding DNA sequence.

### Data availability.

All code and software parameters used to produce the results, as well as ONT quality control reports and phylogenetic matrix, are described in the supplemental material publicly available on GitHub repository Nardonella-NARMHE1-genome (https://github.com/LucPalmieri/Nardonella-NARMHE1-genome). The genome version described in this paper is the first version, and it is under GenBank accession no. JAKMAI010000000. Raw Illumina and Nanopore sequences are available on NCBI Sequence Read Archive under the accession numbers SRR21424116 and SRR20324089, respectively.
